# Can Complementary Prime-Boost Immunization Strategies Be an Alternative and Promising Vaccine Approach Against Dengue Virus?

**DOI:** 10.3389/fimmu.2019.01956

**Published:** 2019-08-27

**Authors:** Iris Valdés, Laura Lazo, Lisset Hermida, Gerardo Guillén, Lázaro Gil

**Affiliations:** Vaccine Department, Center for Genetic Engineering and Biotechnology, Havana, Cuba

**Keywords:** prime-boost immunization, dengue viruses (DENV), recombinant proteins, live-attenuated viruses, antibodies, cell-mediated immune response

## Abstract

Dengue is one of the most important diseases transmitted by mosquitoes. Dengvaxia®, a vaccine registered in several countries, cannot be administered to non-immune individuals and children younger than 9 years old, due to safety reasons. There are two vaccine candidates in phase 3 efficacy trials, but their registration date is completely unknown at this moment. So, the development of new vaccines or vaccine strategies continues to be a priority for the WHO. This work reviews some complementary prime-boost immunization studies against important human pathogens. Additionally, it reviews the results obtained using this regimen of immunization against dengue virus as a potential alternative approach for finding a safe and efficient vaccine. Finally, the main elements associated with this strategy are also discussed. The generation of new strategies of vaccination against dengue virus, must be directed to reduce the risk of increasing viral load through sub-neutralizing antibodies and it must be also directed to induce a polyfunctional T cell response. Complementary prime-boost immunization strategies could emerge as an interesting approach to induce solid immunity or at least to reduce viral load after natural infection, avoiding severe dengue. Subunit vaccine could be safe and attractive antigens for this strategy, especially proteins including B, and T-cells epitopes for inducing humoral and cellular immune responses, which can play an important role controlling the disease.

## Introduction

Vaccination has been the most effective medical intervention to reduce morbidity and death caused by several diseases. Vaccine benefits include the prevention of etiologically confirmed diseases and often extend across the life course of vaccines. Also, vaccines prevent outcomes in the wider community, stabilize health systems, promote health equity, and benefit local and national economies. Vaccination provides stronger broad public health benefits than other preventive and curative interventions (1).

At present, human vaccines are used in the prevention of more than thirty infectious diseases (2, 3). However, there are several pathogens that could not be prevented by vaccination and there are others for which vaccines are not yet available. Some of them are hepatitis C virus (HCV), dengue virus, respiratory syncytial virus, cytomegalovirus, group B *Streptococcus, Staphylococcus aureus*, and *Pseudomonas aeruginosa* (4). In the last century innovative technologies have allowed the development of novel vaccines targeting several diseases or new target populations (5). Among different vaccine modalities, prime-boost immunization strategies could enhance the immunity in the host (6–8).

A prime-boost immunization strategy can be defined as a regimen of immunization with the same immunogen during the prime and booster doses or a regimen of priming the immune system with an immunogen and then boosting with a different immunogen. Several factors including the selection of target antigens, platforms of delivery, routes of immunization, doses, adjuvants, the order of antigens injections, and the intervals between different vaccinations influence the outcome of prime-boost immunization approaches (6–8). The main objective in using this approach is to develop greater levels of immunity compared to the immune response obtained by a single vaccination or by inoculations with the same antigen. Additionally, this approach pursues to elicit both humoral and cellular immune responses, to induce a long-lasting immunity and to induce immunity in mucosal surfaces, in case of some pathogens (6, 9, 10).

Dengue is a mosquito-transmitted viral infection of high incidence worldwide (11, 12). It is caused by four anti-genetically related but distinct dengue virus (DENV) serotypes belonging to the family *Flaviviridae*, genus *flavivirus* (13). These pathogens are estimated to cause up to 390 million infections and 20,000 deaths annually around the world (14). DENV are transmitted mainly by *Aedes aegypti* mosquitoes, and the infection results in a range of clinical outcomes: asymptomatic (most common) or mildly symptomatic illness, uncomplicated dengue fever, or more severe disease including plasma leakage, hemorrhage, and vascular collapse (dengue hemorrhagic fever/shock syndrome) (15, 16). Taking into account the high incidence of the disease, vaccines should be the main approach for controlling dengue epidemics. However, the pathway to developing an effective vaccine is a complex challenge. The main obstacles have been the lack of suitable animal models, the necessity of a tetravalent formulation to protect against each viral serotypes and the lack of a correlate of protection (17). Until a surrogate or correlate of protection is established, efficacy trials of dengue vaccines will need to be conducted based on clinical endpoints, following the virologically-confirmed dengue cases of any severity due to any serotype (18). Moreover, the induction of short-term protection or waning immunity constitutes a big problem because vaccine-recipients can become susceptible to developing severe dengue during a natural infection.

Currently, only three live attenuated tetravalent dengue vaccines (LATVs) have entered or completed phase III clinical trials (19). Only one of them, Dengvaxia®, from Sanofi Pasteur have been approved and licensed in 20 countries (20, 21). The vaccine was obtained by the substitution of the genes that encode for premembrane (prM) and envelope (E) proteins of the attenuated yellow fever virus (YFV) 17D vaccine strain for the prM and E genes of each DENV. These chimeric viruses only induce neutralizing antibodies against the four DENV after three doses given 6 month apart (22). Unfortunately, none or a very low DENV-specific cellular immune response is generated with the vaccine because chimeric viruses lack capsids and the non-structural proteins 3 and 5 of DENV, which are the main targets of CD4+ and CD8+ T-cell responses against these pathogens (23, 24). A recent study conducted with this vaccine demonstrated that the rates of hospitalization among dengue-seronegative vaccinees were higher in the vaccine group than in the control (25). These findings support the hypothesis that Dengvaxia® partially mimics the primary infection in dengue-naïve individuals and increases the risk of severe dengue during a subsequent infection, similar to the risk observed after natural secondary infections. This fact gives important insights into the understanding of dengue protective immunity and it has generated questions for the next outcomes of dengue vaccine efficacy trials (26).

On April 2018, the Strategic Advisory Group of Experts on Immunization recommended that countries, which are considering the introduction of Dengvaxia® to control dengue infection, must conduct a pre-vaccination screening and only dengue-seropositive persons should be vaccinated. Moreover, vaccination should be considered as part of an integrated dengue prevention and control strategy together, along with a well-executed and sustained vector control (27). In addition, seronegative travelers from non-dengue endemic countries should not receive this vaccine, because the vaccine could predispose them to more severe dengue if they were exposed to a natural infection (28).

Another LATV in phase III clinical trial was developed by the National Institute of Allergy and Infectious Diseases (NIAID). This vaccine combined the molecular attenuation of DENV by deletions in the 3′ untranslated region and structural gene chimerization. This vaccine candidate is well-tolerated in humans and induces neutralizing antibodies and a cellular immune response, after only one dose. Also, this vaccine protects volunteers after challenge with partially-attenuated strains of DENV-2 or DENV-3 (29). However, it provokes rash in more than 60% of those vaccinated (30). The third vaccine candidate in phase III clinical testing is based on the attenuated strain of DENV-2 PDK-53 and only contains the capsid and the non-structural proteins of this serotype (31).Therefore, vaccination with this candidate, developed by Takeda, could, in principle, limit the generation of a broad cell-mediated immunity.

One of the attractive alternatives to solving the main disadvantages of LATV (reactogenicity and low efficacy profiles) could be the use of a complementary prime-boost strategy based on combinations of different antigens during the immunizations. These combinations have potential advantages in dengue field, because they could improve the immunogenicity and/or protection against the four DENV. Also, they could reduce the number of doses or the time between doses during immunization schedules. On the other hand, the use of this regimen of immunization could help to induce a balanced immune response against the four serotypes. This review discusses the recent advances in complementary prime-boost immunization strategies used during dengue vaccine development, the main results obtained, as well as the benefits and limitations of these strategies.

## Some Lessons Learned From Complementary Prime-Boost Immunization Strategies

Prime-boost immunization strategies have been used for assessing vaccine candidates against several infectious agents. One of the most studied is the human immunodeficiency virus (HIV). Different types of prime-boost immunization regimens have been evaluated employing DNA, live viral vectors, and recombinant proteins [for recent and extensive reviews see (8, 32)]. Vaccine candidates against HCV, human papilloma virus (HPV) and Ebola virus, among others, have also been extensively evaluated in preclinical and clinical trials using complementary prime-boost immunization strategies (8).

Several important issues influence the outcome of prime-boost immunization regimen of a vaccination. One of the most important is the type of vaccine that will be combined to induce the adequate immune response. Subunit vaccines usually induce humoral immune response while recombinant live vectors, DNA vaccines, and live attenuated viruses stimulate the induction of cell-mediated immunity. Moreover, the pre-immunity against vaccine vectors should be taken into account when designing the immunization schedule. The number of doses, time between them, and the route of immunization and adjuvants, could also affect the results (8).

A complementary prime-boost strategy will be commercially feasible, if the combined regimen induces a significantly greater efficacy profile over single modality vaccines, in order to balance the increased costs and complexities associated with the develop of two antigens, including potential regulatory and licensing problems, as well as logistical hurdles regarding the delivery of vaccines in the field.

Priming with DNA and boosting with viral vectors is an approach focused on the induction of cellular immune responses. Viral vectors usually include adenovirus, vaccinia, fowlpox, and vesicular stomatitis virus (33, 34). It has been proposed that strategies that involve primary vaccinations with DNA followed by boosting with a recombinant poxvirus vector encoding the same immunogen, could elicit a protective CD8+ T cell response in animal models against various diseases such as HIV, malaria, and even cancer (35). In the specific case of malaria control the studies conducted by Li and coworkers were the pioneers demonstrating that the immunization of mice with a recombinant vaccinia virus, after priming with an influenza virus expressing the same CS protein of the parasite, enhances the effectiveness of the anti-parasite immunity, apparently by expanding the antigen-induced CD8+ T cells. The sequence in which these vectors were used for immunization was crucial, because when the reverse immunization was evaluated the results were completely different (36). Although a partial protective immunity against this parasite can be induced by a single immunization with a vaccinia virus presenting the CS protein a second dose with the same antigen failed to enhance the immune response, probably because the primary immune response neutralized the second dose of the virus. Similar results were observed by Rodríguez and colleagues when evaluated the effectiveness of several recombinant influenza and vaccinia viruses to induce a malaria-specific immune response (37). Their results support the concept that live viral vectors expressing the appropriate proteins and/or epitopes can be used as vaccine candidates in prime-boost strategies. Other studies indicate that complementary prime-boost immunization strategies that use recombinant vaccinia virus and bacterial plasmids could be useful for the control of flavivirus diseases (38).

A specific prime-boost strategy has also been employed against HCV. Chronically infected patients were primed with a DNA vaccine expressing the proteins NS3/4A from the HCV genotype 1a and later boosted with a modified vaccinia virus Ankara expressing the proteins NS3/4/5B from genotype 1b. This strategy induced high levels of CD8+T cells and shifted the immune response toward a Th1 pattern (39). A similar response was observed after a prime-boost strategy that used a DNA vaccine, containing the core protein of HCV, followed by the immunization with recombinant Lambda bacteriophage nanoparticles encoding HCV core antigen (40). Both studies suggest that different combinations could induce the required/desired immune response; therefore, the best regimen of immunization will be selected taking into account the troubles faced during the development and/or production of antigens, the prices for their obtaining, etcetera.

Priming with DNA and boosting with proteins (or peptides) is another approach to inducing both humoral and cellular immune responses focusing on the induction of protective antibody responses in non-human primates and more significantly, in humans (7). There are some reports about the efficacy of this strategy against different viral diseases, such as: HCV, HSV, HIV, and HPV (8).

Complementary prime-boost immunization strategies have also been developed to improve the CD8+ T cell response, priming with DNA, or virus like particles (VLP) and boosting with live-vectors. DNA or VLP are able to drive epitopes into the MHC class I pathway and live-vectors enhance the immune response, expressing large amounts of antigen, and stimulating a pro-inflammatory response. Moreover, the boost with VLP has several advantages vs. the boost with a single recombinant protein: (a) to present envelope antigens in their native form; (b) to facilitate the uptake by professional APCs; (c) to activate the endogenous as well as exogenous pathways leading to the presentation of viral antigens by both MHC class I and class II molecules (41, 42).

In the case of HIV, many complementary prime-boost immunization strategies have been evaluated and many of them are based on the priming with a live vector and boosting with a protein. However, this approach needs some improvements to achieve a protective efficacy. A potential solution could be the use of better adjuvants to enhance the immunogenicity of the protein and to improve the duration of the immune response. However, the prime-boost immunization strategy is still an alternative approach for finding a cure against this important pathogen. For many years, research was focused on the induction of humoral immunity as the main arm of the adaptive immune response for a successful vaccine, but cellular immune responses have emerged as a key arm against the infected cells and viral reservoirs (43).

Up to now, the most successful vaccines against microbial pathogens have depended on humoral immunity to achieve protection or even sterile eradication. However, the intracellular bacteria *Mycobacterium bovis (M. bovis)*, which produces tuberculosis (TB), is able to resist most antibody-mediated antibacterial effects by growing inside macrophages (44). Thus, the induction of a cell-mediated immune response to kill the infected cells is crucial to develop an anti-TB vaccine.

Bacille Calmette– Guérin (BCG), the attenuated form of *M. bovis*, has been used for more than 80 years to protect children against severe forms of TB (45). However, its protective efficacy against pulmonary TB varies from 0 to 80% in adults (46); therefore, a more effective vaccine is needed. Prime-boost could be a good strategy for inducing long-term protection combining BCG with other antigens (47).

Although BCG is a strong inducer of Th1 responses, the incidence of TB increases with the time after the first immunization (48), suggesting that the wane of the immunological memory after BCG vaccination is one of the causes of its low protective efficacy. However, this waning immunity cannot be avoided by revaccination (49); therefore, prime-boost immunization strategy could be an attractive approach post-vaccination with BCG. Complementary prime-boost vaccination strategy is known to be highly effective for enhancing anti-TB T cell-mediated immunity (50). The waning immunity constitutes an additional factor to consider prime-boost strategies as promising vaccine alternatives. In the specific case of viruses, attenuated vaccines could have the same disadvantage. In that case complementary prime-boost approaches could be a potential solution.

## Immunological Mechanisms Associated With Complementary Prime-Boost Immunization Strategies

Could a complementary prime-boost immunization strategy be more effective than a prime-boost counterpart, which uses the same antigen during the immunizations? Unfortunately, this question remains “unanswered.” However, there are several examples pointing out that the combination of different antigens can be a promising and interesting approach. Sequential immunizations with different viral vectors generate high levels of CD8+ T-cells and Th1-type CD4+ T-cells in comparison with the response achieved after the boosting with the same antigen (51). Nevertheless, it is not completely obvious which will be the best combination or the viral vector for a specific antigen. In general, the selection of the best option is an empirical process, since it is not possible to evaluate all combinations, due to logistic or economic restrictions.

Some studies propose a rational view to undertaking a complementary prime-boost immunization regimen, when a DNA vaccine is used in the immunization schedule. Usually, a DNA vaccine is employed for priming while recombinant proteins or peptides, inactivated vaccines, or viral vectors are used for boosting the immune response (4). On the other hand, viral vectors are normally used during the first immunization (prime) and the immune response could be boosted with recombinant proteins (4). Moreover, the immunogenicity of this immunization regimen can be improved including other factors that enhance the effect of vaccines such as cytokines (44) and other presentation forms, for example, nanoparticles, or microparticles-based formulations or VLP.

Another important issue to take into account is the type of infection that must be prevented: viral, bacterial, fungal, or parasitical. An effective vaccine requires the induction of different humoral and cellular immune effector mechanisms. The unknown or misunderstood pathogenesis associated with the infecting organism, the absence of good animal models, and also the lack of correlates of protection are additional factors that have hampered the development of vaccines against pathogens like dengue, *Helicobacter pylori*, rotavirus, HIV, *Toxoplasma gondii*, campylobacter, cryptosporidium, and others.

*Helicobacter pylori*, for example, causes a range of diseases including gastric and duodenal ulcers, gastric mucosal associated lymphoid tissue lymphoma, and gastric adenocarcinoma. Unfortunately, none of the antibiotic treatments used by clinicians have eradicated the bacteria (52). Recent results suggest that the induction of a Th2 immune response against the bacteria, limiting the induction of antibacterial antibodies associated with the pathogenesis of the diseases, could control the infection (52). Different approaches must be evaluated and one of them can be a prime-boost immunization strategy. For example, the combination of immunodominant antigens from the bacteria with altered ligand peptides from the heat shock protein 60, an autoantigen associated with the immune-inflammation (53) provoked by the infection, can be an attractive prime-boost alternative. In addition, the combination of mucosal and parenteral immunizations (co-immunization) could be useful to induce a proper immune response and also to disrupt the bacteria-induced tolerance. Prime-boost immunization strategy could be analyzed to control the diseases produced by the list of pathogens mentioned above, although for some of them this strategy has already been evaluated (8).

All licensed vaccines work mainly through the induction of antibodies, and most vaccines approved in the last years have serological markers as immune correlates measured by validated assays. The immune system is redundant and many of its components play pleiotropic functions that can contribute to the protective response against pathogens (17). Antibody functions, such as neutralizing activity, cytotoxicity and opsonophagocytosis may contribute to the protection induced by vaccine candidates. Additionally, CD4+ T cells can activate B and CD8+ T cells to promote inflammation, release cytokines, and lysis infected cells, constituting another arm of the adaptive immune system to control the pathogens (10, 54). The design of the prime-boost immunization strategies will depend on the features of the disease produced by the microorganism and the adequate immune responses to fight against them. Furthermore, several questions should be considered in prime-boost immunization strategies, such as: What type of memory immune response is suitable for the pathogen (central vs. effector, systemic vs. mucosal)? Which protocol will be effective for developing this type of memory immune response? Which routes of administration, number of doses would be adequate? Unfortunately, these and other questions must be addressed during the experimentation.

## Complementary Prime-Boost Immunization Strategies For The Development Of Dengue Vaccines

In the specific case of DENV, prime-boost immunization strategies should be directed to induce high levels of neutralizing antibodies with the main aim to avoid the antibody-dependent enhancement (ADE) of infection and also to induce and/or boost a potent cell-mediated immune response for controlling infected cells. In addition it has been demonstrated that a polyfunctional T-cell response could control the viremia produced under ADE conditions (23, 55), although there is no evidence that T cells are a clear correlate of protection in human. Taking into account epidemiological data, showing that the infection with one serotype confers lifelong immunity against the infective serotype (56), vaccination of a naïve individual must induce both arms of the adaptive immune response. However, a vaccine candidate or vaccine strategy that will induce a potent cell-mediated immunity could have a good efficacy results (57). In contrast, in DENV-immune individuals, vaccination must be addressed to improve the previous immunity, as it has been observed with Dengvaxia.

Multiple vaccine candidates have been developed against DENV, each one with its advantages and disadvantages, but unfortunately there is no a safe and efficacy vaccine against DENV neither antiviral drugs for the treatment of infected patients. From the disappointed results obtained with Dengvaxia® several concerns have arisen about the rationality of many vaccine designs previously tested. For many years researchers have proposed the development of neutralizing antibodies as the main response to achieve protection against DENV, but the lack of correlation between this response and protection observed in animals models and human (58, 59) has challenge this assumption. No protection against DENV-2 was observed in individuals vaccinated with Dengvaxia during a phase IIb clinical trial, despite the induction of neutralizing antibodies against this virus serotype (59). On the other hand, there are growing data demonstrating that the cell-mediate immune response can play a crucial role controlling and reducing the viral load (55, 60–63). This issue has opened the door for finding new vaccine candidates or immunization strategies against this pathogen. Although prime-boost immunization strategies have been previously evaluated against this virus in animal models, none of them have demonstrated to be completely efficacious. However, a prime-boost regimen could be the solution for this complex and threatening disease. Several regimens have been evaluated combining non-replicative antigens and/or replicative vectors in animal models (Table 1). A number of examples will be commented in the following sections. Additionally, in Table 2 we describe the clinical trials posted in the website: https://clinicaltrials.gov, using prime-boost strategies for the immunization. Unfortunately, only one of them has been published.

**Table 1:** Prime-boost strategies used in preclinical studies to develop dengue vaccines.

**Year**	**Prime**	**Boost**	**Regimen**	**Route of inoculation**	**Total number of doses**	**Antigen**	**Immune response**	**Preclinical (animal model)**	**References**
2001	DNA	Recombinant protein	DNA/protein/protein	DNA (intradermally) and protein (intramuscularly)	3 doses	**Prime**: DNA encoding prM and E from DENV-2 **Boost**: DIII of E protein from DENV-2 linked to maltose-binding protein	High antiviral antibodies, low neutralizing antibodies	Mice	(64)
	Recombinant protein	DNA	Protein/DNA/DNA		3 doses	**Prime**: DIII of E protein from DENV-2 linked to maltose-binding protein **Boost**: DNA encoding prM and E from DENV-2	Low antiviral antibodies, long-lasting neutralizing antibodies		
	DNA + recombinant protein	DNA + recombinant protein	Combination		3 doses	**Prime**: DIII of E protein from DENV-2 linked to maltose-binding protein + DNA encoding prM and E from DENV-2 **Boost**: DIII of E protein from DENV-2 linked to maltose-binding protein + DNA encoding prM and E from DENV-2	High antiviral antibodies, long-lasting neutralizing antibodies		
2005	DNA	Protein	DNA/protein	DNA (intramuscularly) and protein (intradermally)	2 doses	**Prime**: DNA encoding E protein + DNA encoding NS1 from DENV-2 **Boost**: recombinant protein E-GST + recombinant protein NS1-GST	Antiviral and neutralizing antibodies	Mice	(65)
2006	DNA	Protein	DNA/protein/protein	DNA (intradermally), inactivated virus and protein (intramuscularly)	3 doses	**Prime**: DNA encoding prM and E from DENV-2 **Boost**: domain III of E protein from DENV-2 fused to maltose-binding protein	Antiviral and neutralizing antibodies, no protection	Monkeys	(66)
	DNA	Inactivated virus	DNA/virus/virus		3 doses	**Prime**: DNA encoding prM and E from DENV-2 **Boost**: inactivated DENV-2 (S16803)	Antiviral and neutralizing antibodies, no protection		
	DNA + recombinant protein	DNA + recombinant protein	Combination		3 doses	**Prime**: DNA encoding prM and E from DENV-2 + domain III of E protein from DENV-2 fused to maltose-binding protein **Boost**: DNA encoding prM and E from DENV-2 + domain III of E protein from DENV-2 fused to maltose-binding protein	Antiviral and neutralizing antibodies, no protection		
	DNA + inactivated virus	DNA + inactivated virus	Combination		3 doses	**Prime**: DNA encoding prM and E from DENV-2 + inactivated DENV-2 (S16803) **Boost**: DNA encoding prM and E from DENV-2 + inactivated DENV-2 (S16803)	Antiviral and neutralizing antibodies, low anamnestic antibody response, no protection		
2007	Adenovirus	DNA	Adenovirus/DNA	Adenovirus (intraperitoneally) and DNA (intradermally)	2 doses	**Prime**: recombinant adenovirus encoding a chimeric domain III of E protein from DENV-2 and DENV-4. **Boost**: DNA vector encoding the same chimeric bivalent antigens	Neutralizing antibodies and T cell response against to both DEN serotypes 2 and 4	Mice	(67)
2007	DNA	Virus replicon particle	DNA/virus replicon particle	Both intramuscularly	3 doses	**Prime**: DNA vaccine encoding prM and E proteins **Boost**: Virus replicon particle of the Venezuelan Equine Encephalitis virus	Neutralizing antibodies, T cell response and protection to challenge	Monkeys	(68)
2007	Recombinant measles vaccine (MV) - EDIII-ectoM	DENV-1	Attenuated vaccine/infective virus	Both intraperitoneally	2 doses	**Prime:** MV-EDIII-ectoM **Boost:** infective DENV-1	High neutralizing antibodies	Mice	(69)
2010	DNA	Protein	DNA/DNA/DNA/ protein	DNA (intramuscularly) and protein (intradermally)	4 doses	**Prime**: plasmid encoding domain II and III of E protein and a fragment of NS1 from DENV-2 **Boost**: recombinant protein E-GST + recombinant protein NS1-GST	Low neutralizing antibodies	Mice	(70)
2010	Tetravalent inactivated virus	Tetravalent live attenuated virus	Inactivated virus/live attenuated virus	Inactivated virus (intramuscularly), DNA (intramuscularly) and live attenuated virus (subcutaneously)	2 doses	**Prime**: tetravalent purified inactivated virus **Boost**: tetravalent live attenuated virus	Neutralizing antibodies and protection	Monkeys	(71)
	Tetravalent DNA vaccine	Tetravalent live attenuated virus	DNA/DNA/live attenuated virus		3 doses	**Prime**: tetravalent DNA encoding prM and E genes from each serotype **Boost**: tetravalent live attenuated virus	Neutralizing antibodies, no protection against DENV-3 challenge		
2010	PD5 + CPS-A	Infective virus	Protein/infective virus	Both subcutaneously	5 doses	**Prime**: PD5 + CPS-A **Boost**: infective virus	Neutralizing antibodies similar to those detected in the group receiving two doses of live virus	Monkeys	(72)
	Infective virus	PD5 + CPS-A	Infective virus/protein		2 doses	**Prime**: infective virus **Boost**: PD5 + CPS-A	Neutralizing antibodies and cellular immune response		
2011	DNA	Vaccinia	DNA/vaccinia	Intramuscularly	2 doses	**Prime**: plasmid encoding E protein from DENV-2 **Boost**: vaccinia virus expressing E protein from DENV-2	Highest levels of E-specific IgG in groups immunized with vaccinia virus, but short duration High production of cytokines by CD4+ T cells in the group inoculated with adenovirus/DNA High CTL killing activity in groups vaccinated with vaccinia/DNA	Mice	(73)
	DNA	Adenovirus	DNA/adenovirus		2 doses	**Prime**: plasmid encoding E protein from DENV-2 **Boost**: adenovirus expressing E protein from DENV-2			
	Vaccinia	DNA	Vaccinia/DNA		2 doses	**Prime**: vaccinia virus expressing E protein from DENV-2 **Boost**: plasmid encoding E protein from DENV-2			
	Vaccinia	Adenovirus	Vaccinia/adenovirus		2 doses	**Prime**: vaccinia virus expressing E protein from DENV-2 **Boost**: adenovirus expressing E protein from DENV-2			
	Adenovirus	DNA	Adenovirus/DNA		2 doses	**Prime**: adenovirus expressing E protein from DENV-2 **Boost**: plasmid encoding E protein from DENV-2			
	Adenovirus	Vaccinia	Adenovirus/vaccinia		2 doses	**Prime**: adenovirus expressing E protein from DENV-2 **Boost**: vaccinia virus expressing E protein from DENV-2			
2011	Infective virus	Recombinant protein	Virus/protein	Subcutaneously	2 doses	**Prime**: infective DENV-2 **Boost**: recombinant protein DIIIC-2	Neutralizing antibodies and cellular immune response	Monkeys	(74)
2017	Infective virus	Recombinant protein	Virus/protein	Subcutaneously	2 doses	**Prime**: DENV-1, DENV-3 or DENV-4 **Boost**: recombinant DIIIC proteins (Tetra DIIIC)	Tetra DIIIC successfully recalled memory B and T cells generated after DENV infection	Monkeys	(75)

**Table 2:** Posted clinical trials evaluating Dengue vaccine candidates in a prime-boost regime.

**Vaccine candidate**	**Status and study's purposes**	**Clinical trials.gov identifier**
**Sponsor:** National Institute of Allergy and Infectious Diseases (NIAID)	**Phase 1:** The purpose of this study is to determine the safety and immune response to a vaccine containing a particular dengue serotype when an individual has been previously vaccinated with a different dengue serotype	NCT00458120 (76)
** Prime --------------- boost**		
rDEN1Δ30 --- rDEN2/4Δ30(ME) (10^3^ PFU)		
rDEN2/4Δ30(ME) --- rDEN1Δ30 (10^3^ PFU)		
rDEN4Δ30------------rDEN1Δ30 (10^3^ PFU)		
rDEN4Δ30 --- rDEN2/4Δ30(ME)(10^3^ PFU)		
**Sponsor:** National Institute of Allergy and Infectious Diseases (NIAID)	**Phase 1:** The purpose of this study is to evaluate the immunologic and clinical response to two dengue vaccines (rDEN1Δ30 and rDEN2Δ30-7169), given 9 months apart, in healthy adults with no history of previous flavivirus infection	NCT02392325
** Prime --------------- boost**		
rDEN1Δ30 --------- rDEN2Δ30-7169 (10^3^ PFU)		
**Sponsor:** National Institute of Allergy and Infectious Diseases (NIAID)	**Phase 1:** The purpose of this study is to evaluate the potential synergistic effect of administering 2 dengue vaccine candidates that were previously shown to be safe and immunogenic in humans. A prime-boost study of tetravalent dengue virus	NCT02239614
** Prime --------------- boost**		
TDENV-PIV (alum) ---180 days-- (TDENV-LAV) (F17)		
**Sponsor:** U.S. Army Medical Research and Materiel Command	**Phase 1:** This is a study of the prime-boost vaccine candidates given in the prime-boost regimen previously demonstrated to have a high level of immunogenicity and immune durability: Day 0 prime (PIV) and Day 180 boost (LAV), and compare it with a previously untested schedule: Day 0 prime (PIV) and Day 90 boost (LAV)	NCT03141138
** Prime --------------- boost**		
TDENV-PIV (alum) ---180 days-- (TDENV-LAV) (F17)		
TDENV-PIV (alum) ---90 days--- (TDENV-LAV) (F17)		
**Sponsor:** U.S. Army Medical Research and Materiel Command	**Phase 1:** This study is being done to evaluate the safety and immune reaction of administering one dose of dengue purified inactivated vaccine and one dose of dengue live attenuated vaccine compared to two doses of inactivated vaccine	NCT03110952
** Prime --------------- boost**		
TDENV-PIV (AS03B) ----- TDENV-PIV (AS03B)		
TDENV-F17 ---------------TDENV-PIV (AS03B)		
TDENV-PIV (AS03B) ------------------ TDENV-F17		

### Combinations of Non-replicative Immunogens

In 2001, Simmons et al. combined a DNA vaccine encoding for pre-membrane (prM) and envelope (E) proteins from DENV-2 and a recombinant fusion protein containing the domain III (DIII) of DENV-2 E protein fused to the maltose-binding protein of *Escherichia coli*. This strategy was tested in BALB/c mice evaluating two combinations (priming with DNA and boosting with the recombinant protein or *vice versa*). Additionally, a group of animals received both antigens at the same time. Both combinations induced a humoral immune response in terms of antiviral and neutralizing antibodies. However, the highest titers of long-lasting neutralizing antibodies were elicited in animals that were co-immunized with both antigens (64). These results suggest that these specific combinations could not be a potential solution against DENV.

Another study combined two plasmids for priming and two recombinant proteins for boosting. DNA plasmids encoded for DENV-2 E and NS1 proteins, respectively, and the recombinant proteins included the same antigens, each fused to the carrier protein Glutathione S Transferase (GST): E-GST and NS1-GST. Both plasmids were co-administrated in BALB/c mice followed by a single dose with both recombinant proteins. The rational design was the inclusion of a broad source of B- and T-cell epitopes, involved in the protective immunity using different regions of the virus. However, this work only evaluated the neutralizing antibody response. The prime-boost immunization strategy showed an increased antibody response to NS1 and E proteins compared to animals that were only vaccinated with the recombinant proteins or DNA constructs (65). Following a similar design, these authors in 2010 reported a second study in mice using similar constructions based on DNA and proteins. They used a new plasmid that included the regions of domain II and III of the E protein and a fragment of NS1 protein. Mice received three administrations of the plasmid encoding for the three viral regions and later were boosted with a single dose of the recombinant proteins GST–E and GST–NS1. As a control, two groups of mice were inoculated only with the parental plasmid or a mix of parental plasmid and both recombinant proteins. Results showed that these combinations were poor immunogenic, with low levels of neutralizing antibody response. Moreover, despite the inclusion of these viral regions, the cellular immune response was not evaluated (70).

Simmons et al. published the first complementary prime-boost immunization study in dengue using non-human primates (66). In this case, they evaluated in rhesus macaques three non-replicating vaccine candidates: a DNA plasmid containing the prM and E genes from DENV-2, a recombinant protein based on DIII of DENV-2 E protein linked to the *E. coli* maltose-binding protein and an inactivated virus of the same serotype. In this study, animals were immunized with three doses using seven different vaccination regimens and the authors measured the humoral immune response and the protective capacity after the homologous virus challenge. All formulations were immunogenic, but the highest neutralizing antibodies titers were detected in monkeys inoculated with three doses of the combinations DNA + protein or DNA + inactivated virus. A similar response was observed in the group receiving three doses of the protein and in animals immunized with three doses of inactivated virus. Unfortunately, despite the good humoral immune response induced in all groups of the study, protection was observed only in animals from the group that received the purified inactivated virus (66); so, the prime-boost immunization strategy did not show an effective response.

An additional study conducted in monkeys (rhesus macaques) combined a recombinant protein that contained a maximum of 60 copies of the DIII of DENV-2 on a multimeric scaffold of *Geobacillus stearothermophilus* E2 (simulating to a VLP) and a DNA plasmid for expressing the DIII portion of the same virus serotype (77). Booth antigens were delivered simultaneously via intramuscular injection (protein) and gene gun. The recombinant protein elicited a robust antibody response to DENV2, with neutralizing antibody responses after the first immunization and reaching high titers following the second and final boosters. Five weeks after the final dose animals were challenged with DENV2. All vaccinated macaques were protected from detectable viremia by infectious assay, while naïve animals had detectable viremia for 2–7 days post-challenge. Although the viral genome was amplified in all animals, no anamnestic antibody response was detected in vaccinated monkeys. This study constitutes an important example of the protective role of neutralizing antibodies elicited against DIII and describes an alternative approach to live-attenuated viruses for potential generation of antibodies against tertiary and/or quaternary epitopes.

### Combinations of Non-replicative and Replicative Immunogens

Other studies have used complementary prime-boost schedules combining non-replicative immunogens and replicative live vectors. This strategy has been used to reduce the troubles associated with replicative vectors, such as the reactogenicity, the anti-vector immunity, and also to improve the immunogenicity associated with the non-replicative immunogens. With this in mind, Simmons et al. reported a study using this approach in rhesus monkeys (71). The study included two experiments; firstly, authors compared the priming capacity of two tetravalent non-replicating immunogens: a tetravalent purified inactivated virus (TPIV) or a tetravalent DNA vaccine (TDNA), separated 2 months of a booster dose with a replicative tetravalent live-attenuated virus (TLAV). Eight months after the booster dose, animals were challenged with an infective strain of DENV-3. As results, all groups generated a humoral immune response against the four serotypes that was measured by ELISA and PRNT. However, complete protection (clearance of virus in sera) after viral challenge was only observed in animals immunized with the TPIV/TLAV regimen. Also, the virus challenge, increased the neutralizing antibodies titters to all serotypes in both experimental groups, but the highest anamnestic immune response was detected in the group inoculated with the TDNA/TLAV combination, where animals were also not protected. An increase of anamnestic immune response after challenge has been associated with a non-protective immune response (78), but we do not completely agree with this as an assumption. Anamnestic response has been observed in non-human primates and humans without detection of viremia (79, 80). For this reason, authors rejected the TDNA vaccine candidate for further experiments. In a second study, the animals were primed with one dose of TPIV and boosted with the TLAV 2 month later, and then animals were challenged with each DENV, 8 months after the last dose. The first inoculation elicited low levels of neutralizing antibodies against the four serotypes, but this response was boosted upon the second inoculation with the TLAV. However, antibodies titers waned gradually until the challenge day, in a serotype-dependent manner. Upon viral challenge, each virus entity was efficiently isolated from sera of animals inoculated with saline solution (acting as control group of the study), with mean duration of viremia of 4.5, 5.0, 4.25, and 2.75 days for DENV-1 to DENV-4, respectively. By contrast, all vaccinated animals were completely protected (71). These results suggest this combination could be a potential prime-boost immunization strategy to deal with a vaccine against DENV.

Another variant used has been the replacement of live-attenuated vaccine by replicative vectors. In this sense, replicative viral vectors, such as those based on adenoviruses or virus replicon particles (VRPs) have demonstrated their usefulness (81). These vectors mimic a live infection by expressing antigens *in situ* after immunization, thereby facilitating the induction of strong T-cell responses, including cytotoxic T lymphocytes. These types of responses are desirable for intracellular and highly variable pathogens and for targeting pathogen-infected cells. Moreover, T-cell responses can target epitopes that are conserved between different strains of the same pathogen (82).

For example, a recombinant adenovirus containing the two DIII regions from DENV-2 and DENV-4 was immunized in mice and later animals were boosted with a plasmid including the identical antigens. This prime-boost immunization strategy showed the induction of neutralizing antibodies and T-cell specific response to both DENV serotypes (67). Unfortunately, this study did not evaluate the protective capacity of this combination but taking into account the immunogenicity, this could be an interesting prospect. Nevertheless, studies conducted in mice have several weaknesses and must be carefully interpreted. Further studies conducted in monkeys with this combination could demonstrate its potentiality, if a protective response is afforded.

In 2013, Azevedo et al. published a study evaluating the immunogenicity and protective efficacy in mice of a pE1D2 DNA vaccine encodes the ectodomain of the envelope DENV2 protein fused to a signal peptide and the YF17D-D2 (constructed by replacing the prM and E genes from the 17D yellow fever vaccine virus by those from DENV-2). BALB/c mice were inoculated with these two vaccines by different prime-boost or simultaneous immunizations. Animals developed neutralizing antibodies and the combined immunization protected against a lethal dose of DENV-2, when compared to each vaccine administered alone. Results also revealed that immunization with the DNA vaccine and the combination with the chimeric virus induced a robust production of IFNγ by CD8+ T lymphocytes (83). Unfortunately, these authors did not evaluate this combination in non-human primates.

In another interesting study a VRP based on Venezuelan Equine Encephalitis virus (VEEV), expressing prM and E proteins from DENV-1, was assayed. The VRP was tested in combination with a DNA plasmid vaccine expressing the identical DENV-1 sequence. One group of Cynomolgus macaques was vaccinated with three doses of DNA plasmid, while a second group received three doses of VRP. The third group of the study was immunized with a combination of DNA plasmid (prime) and VRP (boost), receiving two doses of DNA plasmid and a third dose of the VRP vaccine. Four weeks after the last inoculation, the group immunized with the combination produced the highest virus-binding and neutralizing antibody titters against DENV-1 compared with the other two groups evaluated. Moderate T-cell response was only measured in animals vaccinated with three doses of the DNA plasmid and in the group immunized with the combination of both antigens. When vaccinated animals were challenged with the live virus, all vaccination regimens showed significant protection from viremia, but only animals receiving the combination were completely protected (viremia mean: 0 days) (68). These results highlight the usefulness and potentiality of this strategy as a future vaccine against DENV.

A similar combination was used by George et al. (73). These authors used a prime-boost immunization strategy in mice combining three variants of vaccine candidates: a DNA plasmid, a recombinant adenovirus and a recombinant vaccinia virus. All constructions included the E protein from DENV-2 as immunogen. Animals were primary immunized with each variant and 7 days after, they were boosted with one of the other two antigens. In general, the highest levels in sera of anti-E-specific IgG were observed in mice boosted with vaccinia virus. However, when vaccinia virus was used for priming the levels of anti-E antibodies rapidly decreased. On the other hand, when the cytokines production by CD4+ T cells was measured after *in vitro* stimulation with the E antigen, the results showed a different behavior. Animals from the group primed with the adenovirus and boosted with the DNA plasmid showed the highest secretion levels of IL-2 and IFNγ. In addition, when the CD8+ T cell response was measured by *in vivo* CTL killing activity, the group primed with the vaccinia virus and boosted with the DNA plasmid showed the highest response (73). These results highlight that the pattern of immune response depends on the antigens and also on the order used during the prime-boost immunization strategy. More than one combination must be evaluated to select the best one to induce the proper immune response.

### Combinations of Replicative Immunogens

Several studies have also confirmed the opportunity to combine replicative immunogens in a prime-boost strategy. The first example was proposed by Blander et al. (69). In this work they evaluated the immunogenicity of a dengue vaccine candidate based on a pediatric measles vaccine expressing dengue antigens. The vaccine antigen was obtained by a fusion of DIII of the E protein and the pro-apoptotic ectodomain of M protein from DENV-1. This recombinant construct (EDIII-ectoM) was expressed in a measles vector (MV). The recombinant MV-EDIII-ectoM induced in MV-susceptible mice, a DENV-specific antibody response mainly against the EDIII-ectoM region. These antibodies were able to neutralize the *in vitro* infection produced by DENV-1. In addition, the prime immunization generated a long-term humoral immune response that was successfully boosted when animals were inoculated with a live DENV-1 strain, 9 months after. Unfortunately, the study did not evaluate the protection against DENV-1 in this mouse model (69) and this combination has not been evaluated in non-human primates.

Another example of the combination of replicative immunogens in prime-boost immunization regimens was the study conducted to investigate the effect of the pre-existing immunity to DENV or YFV on the immunogenicity of a tetravalent live-attenuated vaccine in humans (84). In this case, three groups of participants previously inoculated with a monovalent dengue vaccine from DENV-1 or DENV-2 or the yellow fever vaccine were boosted with Dengvaxia®. The results showed that the pre-immune status increased the seroconversion rate and the IFNγ-producing T-cell helper response, upon a single injection with the tetravalent dengue vaccine, particularly against serotypes 1 and 2 (84, 85). Furthermore, viremia in individuals that were primed with the monovalent DENV vaccine was lower than those measured in the group receiving the yellow fever vaccine and in the naïve group. These results suggest that the antibody response generated against DENV-1 or DENV-2 successfully control the viremia produced by the tetravalent vaccine. Additionally, the immune response generated by the non-structural protein from YFV play a role controlling the viremia too. However, the small sample size included in this study constituted a limiting factor in the interpretation of the results; therefore, this study was considered completely descriptive.

### Dengvaxia Protects DENV-Immune Individuals: Could It Be Used for a Potential Prime-Boost Strategy?

Although immunization with Dengvaxia has shown no advantageous results in naïve individuals, long-term follow-up studies have demonstrated that the vaccine is effective in DENV-immune recipients. One possible explanation for this seemingly contradictory result is the presence of higher titers of anti-E antibodies that have undergone affinity/avidity maturation in individuals from the latter group. This is further borne out by the fact that all non-structural viral proteins in Dengvaxia belong to YFV, and thus immunization of DENV-immune individuals with this vaccine is expected to produce very low levels of cell-mediated immune recall. The protective effect of Dengvaxia on DENV-positive vaccinees is, therefore, supposed to rely almost exclusively on the humoral immune response, though this hypothesis remains to be thoroughly verified. This raises the interesting question of whether an inactivated virus vaccine might protect DENV-immune individuals better than live-attenuated Dengvaxia—the answer is probably yes. Since inactivated viruses are adjuvanted and presented to the immune system in the same manner as subunit vaccines, memory B-cells will recognize their target epitopes in the surface of inactivated viruses and turn into plasmatic cells producing protective neutralizing antibodies. From this perspective, Dengvaxia should represent an inferior alternative to an inactivated virus or subunit vaccine, because its replication inside the cells does not stimulate the development of an anti-DENV cell-mediated immune response and thus, any other vaccine candidate expressing DENV-specific B and T-cell epitopes could in theory be engineered to present them to the immune system in a more efficient manner. All said, however, there is something Sanofi Pasteur has definitely taught us, and it is that a prime-boost strategy might represent a promising alternative for the development of anti-DENV vaccines, priming, for instance, with LATV and boosting with subunit vaccines. Boosting doses, of course, are required to maintain protective levels of neutralizing antibodies, and the time between doses would have to be worked out.

In theory, any vaccine candidate inducing high levels of neutralizing antibodies could be administrated before Dengvaxia, which would act as a humoral immune response booster. However, we consider that the induction of cell-mediated immune response is strictly necessary. Some results about this potential combination will be discussed below.

### Tetra DIIIC: A Subunit Vaccine Candidate Against DENV and Its Potential Use in Complementary Prime-Boost Strategies

Experimental prime-boost immunization strategies have been evaluated by a Cuban group that developed a vaccine candidate against DENV based on the DIII of E protein and also the capsid protein of this pathogen. However, in all studies conducted by this group in monkeys, animals have been experimentally infected with DENV. The first study was reported by Valdés et al. combining a non-replicative vaccine candidate with an infective DENV-2. In this experiment, authors primed monkeys with four doses of the recombinant protein PD5 (obtained by the fusion of DIII of the DENV-2 E protein to the C-terminus of protein P64k from *Neisseria meningitidis*) that it was formulated in alum and with the capsular polysaccharide A (CPS-A) from *N. meningitidis* too. Forty-five days after the last immunization animals were infected with a replicative DENV-2 (booster dose), simulating a live-attenuated virus. The results showed a significant increase of anti-DENV-2 antibody response measured after virus inoculation compared with the response measured in non-primed monkeys. In addition, the antibody response was similar to those measured in monkeys inoculated with two doses of the infective virus. In the same study a second group of monkeys was firstly inoculated with one dose of the infective DENV-2 (prime) and 5 months later animals were boosted with one dose of the PD5 formulation. The animals elicited high levels of neutralizing antibodies, which were still detectable for more than 1 year. Additionally, the authors observed that the cellular immune response generated by DENV-2 and measured as IFNγ-secreting cells, was successfully recalled after the recombinant protein administration. Despite the use of an infective virus, this study demonstrated—for the first time—the potential advantage of a prime-boost immunization strategy based on the combination of recombinant proteins and live-attenuated viruses (72). However, none of these studies evaluated the protective efficacy of the combinations.

Two additional studies performed by this group confirm the previous statement. In 2011, these researchers did similar work in monkeys using a new recombinant protein. For this experiment they combined the chimeric protein DIIIC-2 (a fusion protein including DIII region of DENV-2 fused to the N-terminus of the capsid protein from the corresponding serotype) and the infective DENV-2. Animals received one dose of the infective DENV-2 and then were boosted 3 months later with the recombinant protein DIIIC-2. As a result, the animals developed a neutralizing antibody response after the virus infection that was notably boosted after the dose with the chimeric protein DIIIC-2. The neutralizing antibodies induced were long-lasting and a DENV-2-specific cell-mediated immunity was detected 6 months after the booster dose. As conclusion, authors stated that DIII region, when it is properly presented to the immune system, is able to recall a neutralizing antibody response generated by the homologous virus infection in monkeys (74).

**Figure 1:**
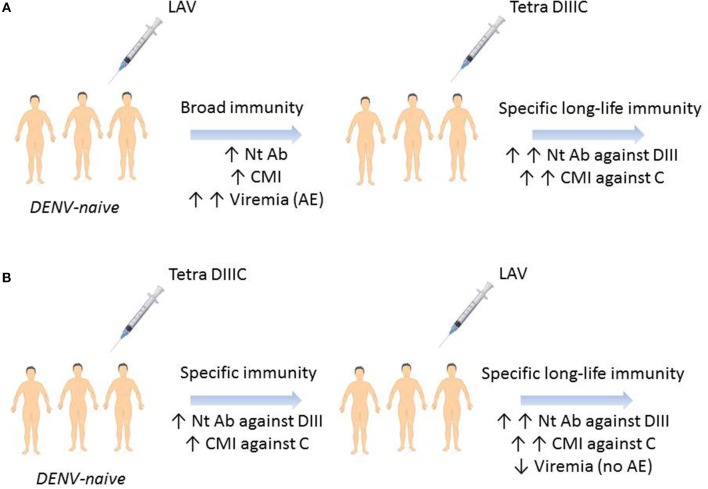
Hypothetical prime-boost immunization regimens outcome for a dengue vaccine. **(A)** DENV-naïve individual immunization with a live-attenuated vaccine will generate humoral and cellular immune responses against all the viral antigens and viremia days with likely adverse events. Boosting with Tetra DIIIC subunit vaccine will stimulate memory B and T cells specific to domain III and capsid protein. **(B)** Tetra DIIIC priming will induce humoral and cellular immune responses against domain III and capsid protein which will control LAVT-booster viremia without adverse events. LAVT, live-attenuated tetravalent vaccine; Nt Ab, neutralizing antibodies; CMI, cell-mediated immunity; DIII, domain III of DENV-envelope protein; C, DENV-capsid protein; AE, adverse events.

Finally, in 2017 Gil et al. reported a last study of prime-boost immunization regimen combining recombinant proteins and infective viruses (75). In this study, rhesus monkeys from Reu Island in Vietnam were experimentally infected with DENV-1, DENV-3, or DENV-4 and 8 month later, the immune response generated by the infection was boosted with one dose of the tetravalent formulation of DIIIC proteins (each one, obtained by the fusion of DIII fused to the homologous capsid protein) (Tetra DIIIC) (86). As it was expected animals developed virus-binding and neutralizing antibodies that were significantly boosted after the administration of Tetra DIIIC. Moreover, the memory IFNγ-producing cells generated by the viruses were successfully recalled by the DIIIC proteins (75). These results highlight the potentiality of Tetra DIIIC as a vaccine candidate against DENV and also its usefulness in prime-boost immunization strategies employing live-attenuated viruses. Obviously, the immunization with live-attenuated viruses will generate memory B and T cells that can be successfully recalled after the administration of Tetra DIIIC, increasing the duration and maturation of the DENV-specific immune response. However, another combination could be also attractive. Priming with Tetra DIIIC can generate an immune response that should reduce the replication of live-attenuated viruses, normally associated with the reactogenicity of this kind of vaccine candidates, without affecting the protective capacity of the combination (Figure 1).

Recently, this group evaluated the combination of Tetra DIIIC with the formulation TV005 (87) developed by the NIH and licensed to the Vietnamese company Vabiotech. Results demonstrate that animals primed with Tetra DIIIC and later immunized with TV005 developed neutralizing antibodies and protective immune responses against the four DENV serotypes. Additionally, the immune response generated by Tetra DIIIC significantly reduced the viremia produced by the live attenuated viruses (88). Although it has been suggested that all four viruses of TV005 vaccine must produce measurable viremia to ensure the induction of homotypic immunity, there is ample data demonstrating that this formulation induces neutralizing antibodies against the four DENV serotypes in >90% of those vaccinated in the absence of detectable viremia (30, 87, 89). Thus, the drop in TV005 vaccine replication produced by the previous administration of Tetra DIIIC has obvious and important clinical implications, as it implies that Tetra DIIIC may represent a potential solution to the reactogenicity problems that have plagued the NIAID's vaccine candidate (30, 90). Further studies should be conducted to evaluate new combinations of these vaccines, as a prime-boost strategy in the reverse order might improve the maturation and duration of the immune response against the four DENV serotypes.

## Conclusions

Despite the introduction of Dengvaxia®, a vaccine against DENV, the WHO has only recommended the introduction of the vaccine in geographic areas with high burden of disease. Moreover, this vaccine cannot be administered in children younger than 9 years old, because it has been demonstrate the increase of hospitalization risk in this age group (91), an important susceptible group, specially infant born from DENV-immune mothers.

Two additional vaccine candidates are in phase 3 clinical trials. Both candidates (NIAID's (formulations TV003 and TV005) and Takeda's candidate contain epitopes for B and T-cell responses against DENV; therefore, the scientific community hopes good results after efficacy trials. However, up to date, the duration and maturation of the immune response elicited by both vaccine candidates is completely unknown and therefore, booster doses could be potentially required. However, booster doses with the same vaccine candidate could be inefficient, as it has been demonstrated with BCG vaccine (49) and even with the NIAID's vaccine candidate (87).

With this scenario and knowing the complexity associated to a DENV vaccine due to the immunopathological phenomena that produce the severe form of the disease, the researchers must find new vaccine candidates or vaccine strategies to control this threatening pathogen. Subunit vaccines could be an attractive alternative, because they are usually safe and can be administered in babies younger than 1-year-old but, unfortunately, they are less immunogenic than replicative immunogens. However, a combination of non-replicative and replicative antigens in prime-boost immunization strategies could be an attractive approach. Obviously, the strategy must induce a long-term safety profile avoiding or controlling the waning immunity and reducing the risk of severe dengue over time after vaccination.

Taking into account the lessons learned from previous studies conducted with DENV and other human pathogens, complementary prime-boost immunization strategy must be addressed to induce high avidity neutralizing antibodies as well as cytotoxic and IFNγ-secreting cells to control circulating microorganism and infected cells. Besides, in the specific case of DENV a good memory B and T-cell responses should be generated, to control the viruses after natural infection. However, factors like immunogens, combinations, order, and time between the immunogens, proper immune responses and others, constitute the main challenges for this strategy.

In the near future, more data on the immunogenicity and efficacy role of prime-boost strategies against DENV will be available. Further studies will be addressed to evaluate potential combinations, schedules of immunization, doses, and timeline between them in order to induce the proper immune response combining both arms of the adaptive immunity, but in our opinion favoring the induction of a potent cell-mediated immune response. All the epidemiological studies conducted and analyzed up to date shown that only 3–5% of secondary heterologous infections produce severe disease manifestations despite the existence of cross-reactive antibodies with the potential capacity to enhance the infection; therefore, prime-boost strategies can be directed to avoid the severe manifestation of the disease, inducing a polyfunctional cell-mediated immunity and efficient neutralizing antibodies.

The Cuban vaccine candidate based on DIIIC proteins could be a potential solution. These proteins form aggregates or particles after the incubation with a synthetic and immunostimulatory oligonucleotide, named ODN 39M. These particles could present quaternary epitopes in their surface for inducing high avidity neutralizing antibodies. Additionally, these proteins contain the capsid region of DENV, which is one of the main targets of cytotoxic and IFNγ-secreting CD4+ T cells, generated during a natural infection. Therefore, combinations of the tetravalent formulation of DIIIC proteins with other replicative or even non-replicative antigens in a prime-boost immunization regimen could be a potential solution for DENV vaccine, which is unsolved today.

## Author Contributions

IV, LL, and LH drafted the review and prepared the figure. GG and LG revised and edited the review.

### Conflict of Interest Statement

The authors declare that the research was conducted in the absence of any commercial or financial relationships that could be construed as a potential conflict of interest.
